# Sequestration and biosynthesis of cyanogenic glucosides in passion vine butterflies and consequences for the diversification of their host plants

**DOI:** 10.1002/ece3.5062

**Published:** 2019-04-13

**Authors:** Érika C. Pinheiro de Castro, Mika Zagrobelny, Juan Pablo Zurano, Márcio Zikan Cardoso, René Feyereisen, Søren Bak

**Affiliations:** ^1^ Department of Plant and Environmental Sciences University of Copenhagen Frederiksberg C, Copenhagen Denmark; ^2^ Department of Systematic and Ecology Federal University of Paraiba João Pessoa Paraíba Brazil; ^3^ Department of Ecology Federal University of Rio Grande do Norte Natal Rio Grande do Norte Brazil

**Keywords:** coevolution, cyanide, *Heliconius*, Lepidoptera, *Passiflora*, specialized metabolites

## Abstract

The colorful heliconiine butterflies are distasteful to predators due to their content of defense compounds called cyanogenic glucosides (CNglcs), which they biosynthesize from aliphatic amino acids. Heliconiine larvae feed exclusively on *Passiflora* plants where ~30 kinds of CNglcs have been reported. Among them, some CNglcs derived from cyclopentenyl glycine can be sequestered by some *Heliconius* species. In order to understand the evolution of biosynthesis and sequestration of CNglcs in these butterflies and its consequences for their arms race with *Passiflora* plants, we analyzed the CNglc distribution in selected heliconiine and *Passiflora *species. Sequestration of cyclopentenyl CNglcs is not an exclusive trait of *Heliconius,* since these compounds were present in other heliconiines such as *Philaethria, Dryas and Agraulis,* and in more distantly related genera *Cethosia *and *Euptoieta*. Thus, it is likely that the ability to sequester cyclopentenyl CNglcs arose in an ancestor of the Heliconiinae subfamily. Biosynthesis of aliphatic CNglcs is widespread in these butterflies, although some species from the *sara‐sapho* group seem to have lost this ability. The CNglc distribution within *Passiflora* suggests that they might have diversified their cyanogenic profile to escape heliconiine herbivory. This systematic analysis improves our understanding on the evolution of cyanogenesis in the heliconiine–*Passiflora* system.

## 1 INTRODUCTION

Land plants have been exposed to herbivores for over 430 million years. To cope with this, plants produce a remarkable diversity of specialized metabolites that act as chemical protections (Fürstenberg‐Hägg, Zagrobelny, & Bak, 2013). In turn, specialist herbivores have evolved under the selection pressure from the chemical defenses of their hosts and adapted to handle their toxicity and even to utilize these metabolites for their own benefit (Nishida, 2014).

The distasteful and colorful butterflies of the Heliconiini tribe selectively feed as larvae on plants from the *Passiflora *genus regardless of the plants’ chemical defenses which, effective against most other herbivores. Due to their larval‐feeding specialization, heliconiines are also called passion vine butterflies. The species diversity (more than 70 heliconiine and 600 passion vines) and multiplicity of feeding guilds found in the heliconiine*–Passiflora* system offer a unique comparative potential to address many intriguing questions on evolutionary and chemical ecology (Gilbert, 1991; Jiggins, 2017). The basal heliconiine genera *Podotricha*, *Philaethria*, *Dryas*, *Dryadula*, *Dione*, *Agraulis,* and *Eueides* (Kozak et al., 2015) are overall generalists, feeding on many *Passiflora* species. In contrast, different degrees of host specialization are observed within *Heliconius*, the most diverse heliconiine genus, with some close phylogenetic associations between infragenic groups of *Heliconius* and their *Passiflora* hosts (Arias et al., 2016; Engler‐Chaouat & Gilbert, 2007). *He* *liconius* species that perform pupal mating (*erato *and *sara‐sapho* groups), an unusual behavior where adult males search for female pupae for mating and either penetrate the pupa or mate the female as soon as it emerges, are characterized by being specialists on *Passiflora* plants of the subgenera *Decaloba* or *Astrophea*. Species of *melpomene*, silvaniforms, and primitive groups (*aoede*, *doris*, *wallacei*), which comprise the nonpupal mating clade, are overall specialists on *Passiflora* species of the subgenus *Passiflora *(Benson, Brown, & Gilbert, 1975).

The chemical defense of the genus *Passiflora* is comprised of different types of cyanogenic glucosides (CNglcs), and the great success of heliconiines feeding on *Passiflora* could be due to the prior ability of these butterflies to biosynthesize CNglcs (Nahrstedt & Davis, 1981). Subsequently, it has been hypothesized that the ability to handle the toxicity of CNglcs was one of the crucial traits that allowed the ancestor of heliconiines to feed on these plants, implying that the ability to detoxify CNglcs preceded their ability to sequester these compounds and perhaps even older than the ability to biosynthesize them (de Castro et al., 2018).

CNglcs are some of the most ancient and widespread defense compounds produced by plants: Whereas other defense compounds are typically restricted to a specific plant group, such as glucosinolates in Brassicaceae, CNglcs are broadly distributed in 2,500 species from ferns to flowering families (Gleadow & Møller, 2014). In insects, CNglc distribution is restricted to a few lineages within Coleoptera and Hemiptera, which seem to obtain these compounds from their diet, and to some lepidopterans (moths and butterflies) where both biosynthesis and/or sequestration is rather widespread (Zagrobelny, Castro, Møller, & Bak, 2018).

**Figure 1:**
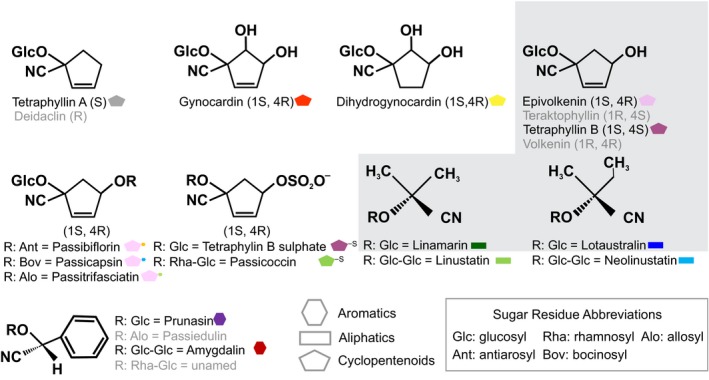
Cyanogenic glucoside (CNglcs) structures reported in *Passiflora* species. Compounds with a gray background were also reported in heliconiines butterflies (Nahrstedt & Davis, 1981; Engler, Spencer, & Gilbert, 2000). Structures with name in gray are enantiomers

CNglcs are glycosylated cyanide‐containing compounds that are not intrinsically poisonous as glucosides. However, tissue damage caused by, for example, herbivore or predator attack leads to CNglcs coming in contact with hydrolytic enzymes (β‐glucosidases and α‐hydroxynitrile lyases), which convert these compounds into toxic hydrogen cyanide (HCN) and aglycones (Pentzold et al., 2017). Protein and nonprotein amino acids are precursors for the biosynthesis of CNglcs and can accordingly be classified as aliphatic, aromatic, or cyclopentenoid (Figure 1). The aromatic and aliphatic CNglcs are derived from protein amino acids like phenylalanine and valine and are broadly distributed in the Plant Kingdom (Zagrobelny et al., 2004). Contrarily, cyclopentenyl CNglcs are synthesized from the nonprotein amino acid cyclopentenyl glycine and have been so far found in five closely related plant families of the Order Malpighiales: Passifloraceae, Turneraceae, Achariaceae, Salicaceae, and Violaceae (Bjarnholt et al., 2008; Tober & Conn, 1985).

The biosynthetic pathway of aromatic and aliphatic CNglcs has been characterized in several plant species. For example, in *Lotus japonicus* the biosynthetic pathway of the aliphatic CNglcs linamarin and lotaustralin consists of two cytochromes P450 (P450s) that convert valine and isoleucine into their respective α‐hydroxynitriles, and an UDP‐glycosyltransferase (UGT) that catalyzes the addition of a sugar residue to these molecules (Takos et al. 2011). In insects, this pathway has been characterized only in the burnet moth *Zygaena filipendulae* which also contain linamarin and lotaustralin. Here, the pathway is composed of the two P450s CYP405A2 and CYP332A3, and UGT33A1 (Jensen et al., 2011). Although the pathways in plants and insects share the same catalytic reactions and enzyme types, the genes encoding the enzymes are not orthologues, confirming that the ability to produce aliphatic CNglcs arose independently in these two Kingdoms. Heliconiine butterflies also synthesize the aliphatic CNglcs linamarin and lotaustralin through the same enzymatic steps as *Zygaena *(Davis & Nahrstedt, 1987), and genes homologous to *ZfCYP405A2* and *ZfCYP332A3* have been found in the *H. melpomene *genome (Chauhan, Jones, Wilkinson, Pauchet, & ffrench‐Constant, 2013) and other *Heliconius* species (Zagrobelny, Castro et al., 2018) although, they have not yet been functionally characterized. This indicates that the biosynthetic pathway of linamarin and lotaustralin has originated in a common ancestor of butterflies and moths (Zagrobelny, Castro et al., 2018).

Some lepidopterans are known to selectively sequester CNglcs from their larval host plant. For example, *Z. filipendulae* sequester linamarin and lotaustralin from *Lotus corniculatus, *possibly to reduce the energetic cost associated with the biosynthesis of these compounds (Fürstenberg‐Hägg et al., 2014). Furthermore, Apollo butterflies (*Parnassius*) are thought to sequester, as well as biosynthesize, sarmentosin, a bitter compound related to aliphatic CNglcs (Bjarnholt et al., 2012). In contrast to these lepidopterans, some *Heliconius* species, especially from the *sara‐sapho* group, have been reported to sequester the cyclopentenyl CNglc epivolkenin from *Passiflora* plants (Engler et al., 2000), a CNglc that differs from the aliphatic CNglc they can biosynthesize. Sequestration of cyclopentenyl CNglcs is also found in other species of the Heliconiinae subfamily, *for example,* in larvae of *Euptoieta hegesia* (tribe Argynnini) which were more cyanogenic when fed on cyanogenic *Turnera ulmifolia* plants (Turneraceae) (Schappert & Shore, 1999; Tober & Conn, 1985). Additionally, *Acraea horta* butterflies (tribe Acraeini) contained the cyclopentenyl CNglc gynocardin when fed on the plant *Kigellaria africana *(Achariaceae), which produces this CNglc (Raubenheimer, 1989). Indeed, most species of the Heliconiinae subfamily feed on Passifloraceae plants and closely related families containing cyclopentenyl CNglcs as larval hosts (Silva‐Brandão et al., 2008).

Remarkably, almost 30 types of CNglcs have been reported in the *Passiflora* genus, and it has been hypothesized that these plants have diversified the structures of CNglcs to specifically evade heliconiine herbivory (Spencer, 1988). However, a detailed comparison between the distribution of the different CNglcs within *Passiflora* and the heliconiine host‐plant preferences has not previously been carried out. Moreover, it is not yet known if basal genera of heliconiines can sequester cyclopentenyl CNglcs from *Passiflora*. The analyses of the cyanogenic potential of *Heliconius* and other heliconiine species have mostly been made with cyanide release measurements (Arias et al., 2016; Cardoso & Gilbert, 2013; Hay‐Roe & Nation, 2007), and this generic technique is unable to show which chemical types of CNglcs are present in each species.

In order to understand the evolution of biosynthesis and sequestration of CNglcs in these butterflies, information regarding the CNglc profiles of heliconiines and their *Passiflora* host are necessary. Here, we investigate the CNglcs profile of selected heliconiines species and combine it with phylogenetic comparisons. Additionally, we overlap the CNglc profile of 42 *Passiflora* species with the butterflies’ host‐plant preferences, in order to elucidate how cyanogenesis has influenced the arms race between heliconiines and their host plants.

## 2 METHODS

### 2.1 Butterfly and plant samples

Pupae from 19 species from the Heliconiinae subfamily were bought from the Costa Rica Entomological Supply (CRES) or Stratford‐Upon‐Avon Butterfly Farm and reared at the greenhouse facilities of the Department of Plant and Environmental Sciences, University of Copenhagen. Pupae were kept in separate cages and maintained under controlled conditions (24–28°C, 80% humidity, 14‐hr light). Cages were inspected every day and emerged butterflies collected in plastic bags, weighed, frozen in liquid nitrogen, and stored at −80°C. All butterflies were collected within 24 hr of eclosion to standardize age, mating history (virgins), and adult food consumption (unfed). *Philaethria dido*, *Philaethria wernickei,* and *Euptoieta hegesia* are not usually bred by butterfly farms, so these species were field‐captured in Brazil, in the Jiqui woods maintained by EMPARN (Parnamirin‐RN) (for more information, see Cardoso & De Lima, 2017) and in forested sites belonging to Miriri Food and Bioenergy S/A (Santa Rita municipality, state of Paraíba). These butterflies were captured with a net and collected in tubes containing 4 ml 80% methanol (v/v).

*Passiflora* samples from this study were from the Copenhagen Botanical Garden and the greenhouse facilities of the University of Copenhagen. Leaves of each species were collected in individual plastic bags, frozen in liquid nitrogen, and kept at −80°C for further analyses.

### 2.2 Methanol extraction

A cold extraction method was used for all butterfly samples as described previously (Zagrobelny, Bak, Olsen, & Møller, 2007). Samples were homogenized with ice‐cold mortars and pestles in 1 or 1.4 ml of a solution containing 55% (v/v) methanol and 0.1% (v/v) formic acid. Samples collected in the field were homogenized in the solution where they were soaked: 4 ml 80% (v/v) methanol. A boiling extraction method (Lai et al., 2015) was used for the plant samples, where leaf pieces of each species were added to microtubes containing 0.5 ml 85% (v/v) methanol and boiled for 5 min in a water bath. All samples were subsequently centrifuged at 10,000 *g* for 5 min and the supernatant filtered (Anapore 0.45 µm, Whatman) to remove insoluble components.

### 2.3 Liquid Chromatography‐Mass Spectrometry (LC‐MS/MS)

Analytical LC‐MS was carried out using an Agilent 1100 Series LC (Agilent Technologies, Germany) hyphenated to a Bruker HCT‐Ultra ion trap mass spectrometer (Bruker Daltonics, Bremen, Germany). Chromatographic separation was carried out using a Zorbax SB‐C18 column (Agilent; 1.8 μM, 2.1 × 50 mm) at a flow rate of 0.2 ml/min. Two solvents were used as mobile phases, A—containing 0.1% (v/v) formic acid with 50 μM NaCl and B—composed of acetonitrile with 0.1% (v/v) formic acid. The gradient program was: 0–0.5 min, isocratic 2% B; 0.5–7.5 min, linear gradient 2%–40% B; 7.5–8.5 min, linear gradient 40%–90% B; 8.5–11.5 min isocratic 90% B; and 11.6–17 min, isocratic 2% B. The flow rate was increased to 0.3 ml/min in the interval 11.2–13.5 min. The oven temperature was maintained at 35°C.

Mass spectral data were analyzed with the native data analysis software. Sodium adducts of tetraphyllin A (RT 5.5 min, [M+Na]^+ ^at *m/z* 294), tetraphyllin B (RT 1.3 min, [M+Na]^+ ^at *m/z* 310), epivolkenin (RT 2.3 min, [M+Na]^+ ^at *m/z* 310), gynocardin (RT 1.4 min, [M+Na]^+ ^at *m/z* 326), linamarin (RT 2.4 min, [M+Na]^+ ^at *m/z *270), lotaustralin (RT 5.5 min, [M+Na]^+ ^at *m/z* 284), prunasin (RT 7 min, [M+Na]^+ ^at *m/z* 317), and amygdalin (RT 6.6 min, [M+Na]^+ ^at *m/z* 480) were detected and their RTs compared to authentic standards (Engler et al., 2000; Jaroszewski et al., 2002; Møller, Olsen, & Motawia, 2016). The total amount of each compound was estimated based on extracted ion chromatogram (EIC) peak areas and quantified based on calibration curves of linamarin, lotaustralin, and amygdalin. Linustatin (RT 3 min, [M+Na]^+ ^at *m/z* 432), dihydrogynocardin (RT 1.4 min, [M+Na]^+ ^at *m/z* 328), tetraphyllin B sulfate (RT 1.3 min, [M+Na]^+ ^at *m/z* 390), passicapsin (6.5 min, [M+Na]^+ ^at *m/z* 440), and passibiflorin (RT 5.8 min, [M+Na]^+ ^at *m/z* 456) were identified by their m/z, fragmentation pattern (MS/MS), and comparison with data reported in the literature regarding these compounds. Quantification of CNglcs present in the butterfly samples was based on a regression equation calculated from a standard curve.

### 2.4 Comparative analyses

MANOVA and pairwise comparisons using Geomorph v.3.0.5 package (Adams, 2014) in R (R Core Team, 2017) were performed to analyze infraspecific (sexual dimorphism) and interspecific differences in CNglc concentrations. Data were not normally distributed; therefore, the analyses were performed with square root transformed data, as well as with the raw data for comparison. Preliminary analyses did not support sexual dimorphism in CNglc composition of the butterflies; thus, female and males were not distinguished in further investigations.

Phylogenetic comparative methods were performed using the tree‐hypothesis proposed by Kozak et al. (2015). *Phytools* 0.6‐20 package (Revell, 2012) in R (R Core Team, 2017) was used for the ancestral reconstruction of CNglc biosynthesis (aliphatic) and sequestration (cyclopentenoids) in heliconiines (*Cethosia cyane* was used as an outgroup). The phylogenetic signal of the butterflies’ CNglc profiles was measured utilizing the *k*_mult_ approach (Adams, 2014), a generalization of the *K* statistic (Blomberg, Garland, & Ives, 2003). Values of *K*_mult_ < 1 imply that taxa resemble each other phenotypically less than expected under Brownian motion, whereas values of *K*_mult_ > 1 imply that close relatives are more similar to one another phenotypically than expected under Brownian motion.

Additionally, to build a phylomorphospace (Sidlauskas, 2008), we performed a principal component analyses (PCA) of the butterflies’ CNglc profiles and projected the first two components on the phylogeny. Both analyses were conducted using Geomorph 3.0.5 package (Adams, 2014) in R (R Core Team, 2017).

## 3 RESULTS

### 3.1 Cyanogenic glucoside distribution within heliconiines

Although all heliconiines were thought to be cyanogenic to some degree, the CNglc composition of only a few species has been reported to date. Therefore, we identified and quantified the CNglcs present in male and female adults of 22 species that belong to the subfamily Heliconiinae.

**Figure 2:**
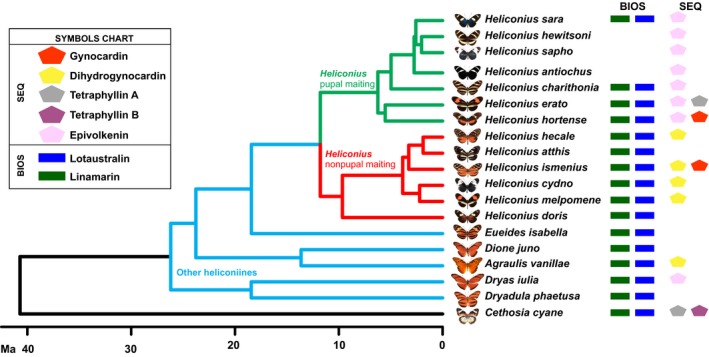
CNglc distribution in the Heliconiini tribe and in the outgroup *Cethosia cyane*. Phylogenetic dendrogram is according to Kozak et al. (2015)

The aliphatic CNglcs linamarin and lotaustralin were found in almost all species analyzed, confirming the widespread occurrence of CNglc synthesis in the tribe Heliconiini (Figure 2). Interestingly, some species of the *sara‐sapho* clade (*H. sapho,* *H. hewitsoni,* and *H. antiochus*), specialized on plants of the *Astrophea* subgenus as larval hosts, lacked these compounds. Instead, these species contained epivolkenin, a compound derived from sequestration because insects are not known to biosynthesize its precursor cyclopentenyl glycine.

Surprisingly, cyclopentenyl CNglcs were not found exclusively in *Heliconius *species as previously reported (Engler‐Chaouat & Gilbert, 2007). They were also present in *A. vanillae*, *D. iulia,* in the outgroup *C. cyane *(Figure 2), and in wild‐caught *Philaethria dido* and *Euptoieta hegesia* (data not shown). These findings indicate that sequestration of cyclopentenyl CNglcs is a broadly distributed trait within the subfamily Heliconiinae and is not a derived trait of *Heliconius*. Besides epivolkenin, the additional cyclopentenyl CNglcs dihydrogynocardin, gynocardin, tetraphyllin B, and tetraphyllin A were found in some species. We have not found earlier reports for the presence of tetraphyllin B, tetraphyllin A, and dihydrogynocardin in butterflies, or of gynocardin in *Heliconius*. Tetraphyllin B is a diastereomer of epivolkenin, whereas tetraphyllin A has a nonhydroxylated cyclopentenoid ring and gynocardin an extra hydroxylation in C5 (Figure 1). Dihydrogynocardin is the only cyclopentenylglycine‐derived CNglc containing a cyclopentanoid ring.

Across all *Heliconius* species, epivolkenin was present in the entire pupal mating clade although only traces of this compound were found in *H. charithonia*. In addition, *H. hortense* and *H. erato *also contained gynocardin and tetraphyllin A, respectively (Figure 2). Epivolkenin was conspicuously absent in the nonpupal mating clade, which almost exclusively sequestered dihydrogynocardin, although gyanocardin was found in *H. ismenius* and *H. atthis,* and *H. doris* did not contain any cyclopentenyl CNglcs.

### 3.2 Total CNglc concentration in heliconiines and host‐plant specialization

**Figure 3:**
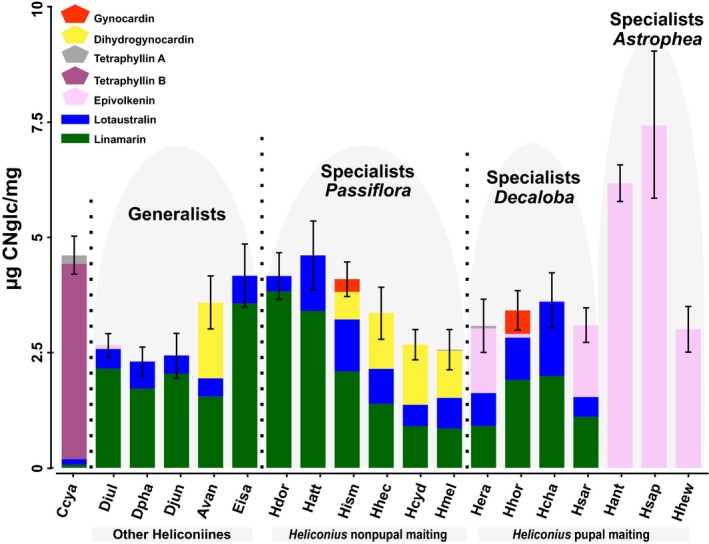
CNglc concentrations in heliconiine species and in the outgroup *Cethosia cyane*. CNglc composition of heliconiine butterflies is also categorized by their larval‐feeding strategy: generalists, *Passiflora *specialists, *Decaloba *specialists, and *Astrophea *specialists

Our results suggest that the CNglc composition of heliconiine butterflies correlates with their larval‐diet specialization (Figure 3). The species with the highest total concentration of CNglcs were *H. sapho *(7.45 µg/mg) and *H. antiochus *(6.17 µg/mg), which are both *Astrophea* specialists and contained only epivolkenin (Figure 3). In the pairwise comparisons between species, the total CNglc concentration in *H. sapho* was significantly higher than most heliconiines, except *Eueides isabela (*4.17 µg/mg) and *H. atthis *(4.61 µg/mg) which contained only the biosynthesized CNglcs linamarin and lotaustralin, and *H. antiochus *(Figure 3 and Supporting Information Table S1). *Heliconius* species that were *Passiflora* and *Decaloba* specialists had similar CNglc concentrations, apart from *H. atthis *that had significantly higher concentrations than *H. melpomene *(2.56 µg/mg). Although *E. isabela *had more linamarin and lotaustralin and *Agraulis vanillae* contained additional cyclopentenyl CNglcs in its composition, there were no significant differences in the total concentration of CNglcs between other heliconiines which utilize many passion vine as larval host (Figure 3 and Supporting Information Table S1). The genus *Heliconius *tend to have more CNglcs than other heliconiine genera although differences were not statistically significant (*F*_1,133 = _4.12, *p* = 0.053).

Overall, there were no significant differences in CNglc concentration between male and female butterflies in this study (data not shown). Nevertheless, sequestration of epivolkenin in *D. iulia* and *H. charithonia* was observed only in female butterflies. Additionally, cyclopentenyl CNglcs were absent in most of the males of *H. hecale *(data not shown).

### 3.3 Biosynthesis of aliphatic versus sequestration of cyclopentenyl CNglcs

**Figure 4:**
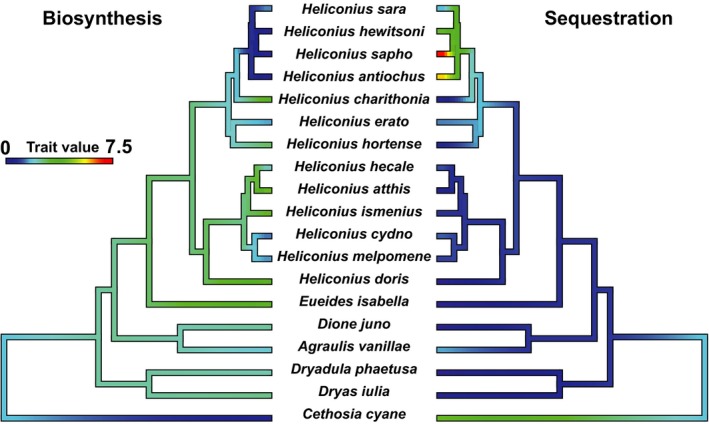
Ancestral reconstruction state of biosynthesis of aliphatic CNglcs and sequestration of cyclopentenyl CNglcs illustrating the balance between these two processes in heliconiines (+*C. cyane*). Trait value refers to the total mean CNglc biosynthesized or sequestered (μg/mg) by species.

The heliconiine species analyzed in this study could be sorted into three groups based on their source of CNglcs: biosynthesis only, sequestration only, or both (Figure 2). The highest CNglc concentrations were found in species that either performed biosynthesis only (*E. isabela*, *H. atthis,* and *H. doris*) or sequestration only (*H. sapho* and *H. antiochus*) (Figure 3). In the *Heliconius* species that used both strategies, we observed a potential trade‐off between these two processes: species that sequestered cyclopentenyl CNglcs tended to have lower amounts of biosynthesized linamarin and lotaustralin (Figure 4). Preferences for biosynthesis or sequestration are apparently phylogenetic related (Figure 4) possibly due to a direct link with larval‐diet specialization (Figure 3).

*Cethosia cyane* and *H. sapho*, *H. antiochus,* and *H. hewitsoni (Heliconius*–*Astrophea* specialists) had significant differences in the concentration of biosynthesized CNglcs compared to the other species. Only traces of linamarin and lotaustralin were found in *C. cyane*, while in *Heliconius*–*Astrophea *specialists, these compounds were absent (Figures 3 and 4, Supporting Information Table S2). Contrarily, *C. cyane* and *Heliconius*–*Astrophea* specialists contained greater concentrations of sequestered CNglcs than all other species.

Within *Decaloba* specialists, *H. charithonia *had significantly higher concentrations of biosynthesized CNglcs than *H. sara* and *H. erato,* but similar to *H. hortensis. *In contrast*, H. erato and H. sara *had comparable concentrations of sequestered CNglcs and differed from all other analyzed species. (Figure 3 and Supporting Information Table S2).

The total concentration of CNglcs did not differ between the *Passiflora* specialists that performed both biosynthesis and sequestration (*H. ismenius*, *H. hecale*, *H. cydno*, and *H. melpomene*) (Figure 3 and Supporting Information Table S2). However, *H. doris* and *H. atthis*, which only biosynthesize linamarin and lotaustralin, have significantly higher concentration of these compounds than the remaining species in the group, except for *H. ismenius*.

There were no significant differences in the concentration of biosynthesized CNglcs within the heliconiine species with generalist‐feeding preferences. Additionally, *Agraulis vanillae *was the sole species showing significant differences regarding CNglc sequestration due to the presence of dihydrogynocardin (Figures 3).

### 3.4 Phylogenetic divergences in the CNglc profile of heliconiines

Among basal heliconiines, CNglcs were obtained mainly through biosynthesis, while sequestration was performed only by some species (Figure 2). The ability to sequester CNglc seems to gain more importance through the *Heliconius* genus until it becomes the sole source of CNglcs in the most specialized species in the *sara‐sapho *group (*H. sapho, H. hewitsoni, H. antiochus*) which are *Astrophea* specialists. CNglc sequestration is also the main strategy performed by the outgroup *Cethosia cyane *(Figure 4), an Asian species of the Heliconiinae subfamily, which does not belong to the Heliconiini tribe.

**Figure 5:**
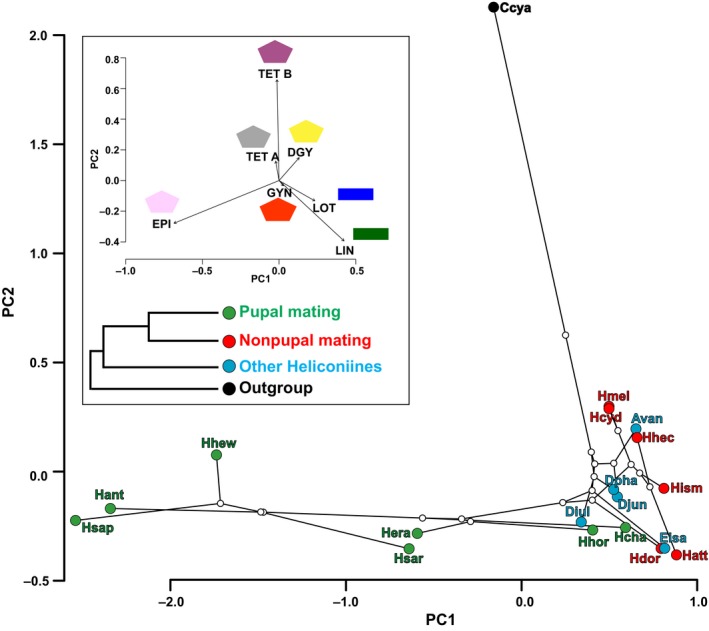
Phylomorphospace correlating the concentration of each CNglcs in heliconiines + *Cethosia cyane* (outgroup) with their phylogenetic distances. Each colored point represents a concentration value of CNglc by species and white points the hypothesized ancestral phenotype. Lines connect related species through hypothetical ancestors. Different colors represent the phylogenetic groups in box. The phylogeny (Kozak et al., 2015) has been pruned to include only species used in our study. Hsap = *Heliconius sapho*; Hant = *H. antiochus*; Hhew = *H. hewitsoni*; Hsar = *H. sara*; Hcha = *H. charithonia;* Hera = *H. erato*; Hhor = *H. hortense*; Hmel = *H. melpomene;* Hcyd = *H. cydno*; Hhec = *H. hecale; *Hatt = *H. atthis; *Hism = *H. ismenius; *Hdor = *H. doris; *Eisa = *Eueides isabella; *Avan = *Agraulis vanillae; *Djun = *Dione juno; Diul = Dryas iulia; *Dpha = *Dryadula phaethusa;* Ccya = *Cethosia cyane*

The phylomorphospace in Figure 5 allows a two‐dimensional visualization of the phylogeny in the morphospace (PCA axes). In this case, we correlated the concentration of CNgcls in heliconiines and the outgroup *Cethosia cyane* with their phylogenetic distances. Sequestration was the main strategy in most pupal mating *Heliconius* species and *C. cyane *(Figure 4), although the compounds obtained by them have a different chemical structure. Most of the pupal mating *Heliconius *species (except *H. hortense* and *H. charithonia*) are on the negative PC1 axis, which correspond to the presence of the sequestered CNglc epivolkenin (Figure 5 and inset). Nonpupal mating *Heliconius* and other heliconiines are in the positive extreme, because they perform biosynthesis of linamarin and lotaustralin and sequestration of other cyclopentenyl CNglcs besides epivolkenin (gynocardin, dihydrogynocardin, tetraphyllin A). *Cethosia cyane* is totally separated from the heliconiines in the positive extreme of PC2 axis, due to its sequestration of tetraphyllin B and its phylogenetic distance. Closely related species are chemically more similar among them than with more distant species (*K*_mult_ = 0.49, *p* = 0.001), supporting the segregation pattern found in the phylomorphospace (Figure 5). Low phylogenetic signal is probably a consequence of chemical resemblances between some *Heliconius* species and basal heliconiines (e.g., *H. charithonia and H. hortense* which are separated in the multidimensional space of the pupal mating group and overlapping with other heliconiines).

### 3.5 Distribution of CNglcs within *Passiflora*

The CNglc profiles of 22 *Passiflora* species were analyzed in order to document which compounds would be available to heliconiine larvae from their host plants. These results are shown in Table 1, together with information compiled from the literature regarding the CNglc composition of 20 other *Passiflora* species.

**Table 1:** CNglcs distribution among *Passiflora* species

Subgenus	Section	Serie	Species	CNglcs	References
*Deidamioides*	*Discophora*		*P. discophora*	TEB	Jaroszewski et al. (2002)
*Astrophea*			*P. amoena*	LIN, LOT, LNT	This work
*Passiflora*	*Stipulata*	*Grannadillastrum*	*P. foetida*	TEA, TEB	This work
			*P. caerulea*	TEB, TEB(S)	This work Jaroszewski and Fog (1989)
			*P. violacea*	TEB(S)	Jaroszewski et al. (2002)
			*P. subpeltata*	LIN	Olafsdottir et al. (1989)
			*P. menispermifolia*	EPI, TEA	This work
	*Passiflora*	*Calopathanthus*	*P. racemosa*	TEB(S)	Jaroszewski and Fog (1989)
			*P. mathewsii*	nd.	This work
		*Passiflora*	*P. incarnata*	GYN	Spencer and Seigler (1984)
			*P. edulis*	PRU, AMY	This work
			*P. cincinnata*	PCP	This work
	*Coccinea*		*P. coccinea*	PCO(S)	Kevin Spencer and Seigler (1985)
			*P. vitifolia*	TEB(S)	This work
	*Laurifolia*	*Tiliifoliaa*	*P. serratodigitata*	EPI, PBF	This work
			*P. platyloba*	PRU, AMY	This work
			*P. maliformis*	PRU, AMY	This work
		*Quadrangulares*	*P. quadrangularis*	EPI, TEA, TEB(S)	Jaroszewski and Fog (1989)
		*Laurifoliae*	*P. ligularis*	nd.	This work
			*P. laurifolia*	nd.	This work
			*P. riparia*	nd.	This work
*Tetrapathea*			*P. tetrandra*	TEA, TEB	Olafsdottir et al. (1989)
*Decaloba*	*Hahmopathanthus*		*P. guatemalensis*	EPI, GYN, DGY	This study, Jaroszewski et al. (2002)
	*Disemma*		*P. herbertiniana*	EPI, TEA	Jaroszewski et al. (2002)
	*Multiflora*		*P. holosericea*	EPI, TEA	This study
	*Auriculata*		*P. auriculata*	EPI, GYN	This study
			*P. jatunsachenis*	EPI	This study
	*Cieca*		*P. coriacea*	EPI, TEB	This study
			*P. suberosa*	EPI, GYN	This study
	*Bryonioides*		*P. morifolia*	LIN, LOT	Olafsdottir et al. (1989)
			*P. pendens*	LIN, LOT	Spencer et al. (1986)
			*P. adenopoda*	LIN, LOT	Spencer et al. (1986)
	*Decaloba*	*Xerogama*	*P. capsularis*	EPI, PCP	Olafsdottir et al. (1989)
			*P. citrina*	PSC	Jaroszewski et al. (2002)
	*Decaloba*	*Decaloba*	*P. lutea*	PBF, LIN, LOT	Spencer and Seigler (1985)
			*P. indecora*	PBF	Jaroszewski et al. (2002)
			*P. kalbreyeri*	PBF	Jaroszewski et al. (2002)
			*P. apetala*	PBF	Jaroszewski et al. (2002)
			*P. trifasciata*	PTF	Olafsdottir, Jaroszewski, & Seigler, (1991)
			*P. biflora*	PBF, PCP	This work, Olafsdottir et al. (1989)
			*P. colivauxii*	PBF, PCP	This work
			*P. cuneata*	PBF	Jaroszewski et al. (2002)
			*P. talamascensis*	PBF	Spencer and Seigler (1985)
			*P. murucuja*	PBF	Jaroszewski et al. (2002)
			*P. perfoliata*	PBF	Jaroszewski et al. (2002)

Note

Aliphatic CNglc: LIN = linamarin; LOT = lotaustralin; LNT = linustatin; Aromatic CNglcs: AMY = amygdalin; PRU = prunasin; CNglcs bisglycosylated with unusual sugars: PBF = passibiflorin, PCP = passicapsin, PTF = passitrifasciatin; nd = CNglcs not detected; Sulphated: PCO(S) = passicoccin; TEB(S) = tetraphyllin B sulfate.

For chemical structure of these compounds see Figure 1.

Linamarin, lotaustralin, and linustatin were found in *P. amoena, *and epivolkenin reported in *P. pittieri*, both from the *Astrophea *subgenus. In addition, tetraphyllin B and tetraphyllin A have been reported in *P. discophora, *which belong to the *Deidaminoides subgenus *(Jaroszewski et al., 2002).

Cyclopentenyl CNglcs were found in the *Passiflora* subgenus, but in most species, these compounds are sulfated. For example, *P. caerulea *and *P. racemosa* have tetraphyllin B sulfate (Jaroszewski & Fog, 1989), and passicoccin, a diglycoside and sulfated version of epivolkenin, was discovered in *P. coccinea *(Spencer & Seigler, 1985). Aliphatic CNglcs seem to be very rare in this subgenus, being reported only in two species. In addition, the aromatic CNglcs prunasin and amygdalin were found in *P. platyloba*, *P. maliformis*, and *P. edulis. *Although all *Passiflora* are thought to be cyanogenic, cyanogenic glucosides were not detected in our analyses in three species of the *Passiflora* subgenus, specifically *P. mathewsii*, *P. laurifolia, *and *P. riparia *(Table 1).

Within the *Decaloba* subgenus, cyclopentenyl CNglcs were present in all species examined, except in the *Bryonioides *section reported to have exclusively aliphatic CNglcs. However, in the *Decaloba* section, the most diverse section of the *Decaloba* subgenus, the cyclopentenyl CNglcs were bisglycosylated (sugar added in two different positions of the aglycone) with unusual sugars, and they seem to be derived from epivolkenin. Passibiflorin is present in several species, including *P. biflora*, and it is glycosylated with an antiarosyl sugar residue. Passicapsin, found in *P. capsularis* and *P. citrina, *has a boivinosyl residue, while passitrifasciatin, which is reported only *in P. trifasciata*, has an allosyl. Simple cyclopentenyl CNglcs (monoglycosides), such as epivolkenin, are present mainly in the basal species of the *Decaloba *subgenus and also in the sister subgenus *Tetrapathea* in *P. tetrandra*.

## 4 DISCUSSION

### 4.1 Cyanogenesis in the Heliconiinae: The chicken and egg paradox

Our results show that sequestration of cyclopentenyl CNglcs is not an exclusive ability of a few *Heliconius* species as previously hypothesized. It is, in fact, widespread, not only among *Heliconius* butterflies, but also across related genera, such as *Dryas*, *Philaethria,* and *Agraulis*. Cyclopentenyl CNglcs were found even in species outside the Heliconiini tribe such as *Euptoieta hegesia* (tribe Argynnini) and *Cethosia cyane*. Accordingly, sequestration of cyclopentenyl CNglcs probably arose in a common ancestor of the Heliconiinae subfamily or even earlier. In fact, most species of the Heliconiinae subfamily use plants from families where cyclopentenyl CNglcs have been reported as larval host plants. The only exceptions are the American genera of the tribe Acraeini, *Actinote,* and *Altinote*, hypothesized to have shifted preference to Asteraceae plants to avoid competition with heliconiines in America (Brown & Francini, 1990).

Since most butterflies of the Heliconiinae subfamily can biosynthesize aliphatic CNglcs and also sequester cyclopentenyl CNglcs from their larval host, the obvious question is—*Which came first in the evolutionary process, biosynthesis or sequestration*?

Whereas sequestration of cyclopentenyl CNglcs has been reported only in butterflies of the Heliconiinae subfamily, biosynthesis of linamarin and lotaustralin has been demonstrated in several species of butterflies and moths belonging to many taxonomically distinct families, such as Zygaenidae, Limacodidae, Heterogynidae, Nymphalidae, and Lycaenidae (Davis & Nahrstedt, 1982; Nahrstedt, 1988; Zagrobelny, Castro et al., 2018). The biosynthetic pathway in the burnet moth *Z. filipendulae* is encoded by the genes *ZfCYP405A2*, *ZfCYP332A3,* and *ZfUGT33A1 *(Jensen et al., 2011) and putative homologues of these P450s have been found in the genome of the postman butterfly *H. melpomene, HmCYP405A4*, *HmCYP405A5, HmCYP405A6, *and *HmCYP332A1 *(Chauhan et al., 2013) and in other *Heliconius* species (Zagrobelny, Jensen, Vogel, Feyereisen, & Bak, 2018). Therefore, it is likely that the ability to biosynthesize aliphatic CNglcs appeared during the early radiations of the Lepidoptera in a common ancestor of butterflies and moths over 150 MYA and was later lost in many lepidopteran species. Assuming that the biosynthesis of aliphatic CNglcs arose in the early radiation of Lepidoptera and sequestration of cyclopentenyl CNglcs in an ancestor of the Heliconiinae subfamily, biosynthesis would be the ancestral trait.

Nevertheless, to confirm this hypothesis it is necessary to identify which adaptations are involved in the sequestration of cyclopentenyl CNglcs by heliconiines and the sequestration of different CNglcs present in other insects. Yu, Fang, Zhang, and Jiggins (2016) demonstrated that larvae of *H*. *melpomene* expressed different sets of transporters when reared on *Passiflora* species with different CNglc profiles, and characterization of these transporters would improve our understanding of the sequestration of cyclopentenyl CNglcs.

A further consideration is the ability to detoxify cyanide to avoid intoxication, which enables both sequestration and biosynthesis of CNglcs in Lepidopterans and most likely predates these two processes. Butterflies, moths, and mites detoxify cyanide using β‐cyanoalanine synthase (CAS) which converts cysteine and HCN into β‐cyanoalanine and H_2_S (Zagrobelny, Jensen et al., 2018). The *CAS* gene is shown to have been horizontally transferred from bacteria to a common ancestor of Lepidoptera, allowing them to colonize cyanogenic plants (Wybouw et al., 2014). Three putative *CAS* genes have been found in the *H. melpomene *genome (Wybouw et al., 2014) emphasizing the importance of cyanide metabolism for these butterflies. It is still not known if the CAS genes are also present in other heliconiines and *Heliconius* species.

### 4.2 The balance between biosynthesis and sequestration of CNglcs

Our results suggest that there is a potential trade‐off between biosynthesis of aliphatic CNglc and sequestration of cyclopentenyl CNglcs in heliconiines (Figure 4). This trade‐off is probably due to the fact that biosynthesizing CNglcs is costlier than sequestering them from the larval host plants. This would explain why some highly specialized species of the *sara‐sapho* group seem to have lost CNglc biosynthesis in favor of epivolkenin sequestration (Figure 2).

This balance between biosynthesis and sequestration of cyanogenic glucosides has also been found in larvae of *Zygaena filipendulae, *which produce linamarin and lotaustralin, as well as sequester them from their host *Lotus corniculatus. *Although larvae of *Z. filipendulae* had similar concentrations of linamarin and lotaustralin when reared on high‐cyanogenic, low‐cyanogenic, and acyanogenic *L. corniculatus*, their growth and body mass at pupation was greatly reduced when these compounds were low or absent in their diet (Zagrobelny, Bak, Ekstrøm, Olsen, & Møller, 2007). This indicates that *Z. filipendulae* larvae increase CNglc biosynthesis when these compounds are not available in their host to be sequestered, and that biosynthesis is costly for their development. Similarly, the wings of *Heliconius erato* vary in size according to their larval host plant (Jorge, Cordeiro‐Estrela, Klaczko, Moreira, & Freitas, 2011) which could be a consequence of the energy expended on CNglc production due to the amount of these compounds available in their diet.

Moreover, Fürstenberg‐Hägg et al. (2014) demonstrated that *Z. filipendulae *larvae upregulate gene expression of its CNglc biosynthetic pathway (*CYP405A2*, *CYP332A3,* and *UGT33A1*) when these compounds are not available in their host plant. CYP405A2, the first enzyme of the pathway, is controlled at both transcriptional and enzyme steady state level by the concentration of linamarin and lotaustralin in the diet *of Z. filipendulae *and regulates the intensity of biosynthesis. Contrary to *Z filipendulae*, heliconiines sequester different kinds of CNglcs from their larval host than they can biosynthesize, so further investigations are needed to understand how the interplay of these two processes is regulated in these butterflies.

### 4.3 Evolution of cyanogenesis in heliconiines is associated with their larval host specialization

Across all species analyzed, *H. sapho* had the highest total concentration of CNglcs. This is in line with a previous report by Engler‐Chaouat and Gilbert (2007), who also found that *H. sapho,* and closely related species, were the most cyanogenic species within the *Heliconius *genus. Similar to *H. sapho*, all species of the *sara‐sapho *group feeding on *Astrophea* plants contain only epivolkenin, and it is possible that they may have lost the ability to synthesize aliphatic CNglcs to become more specialized in sequestration as suggested by Engler‐Chaouat and Gilbert (2007). Yet, apparently functional P450 genes associated with the biosynthesis of linamarin and lotaustralin are present in the genome of *H. sapho *(Zagrobelny, Castro et al., 2018) suggesting that the lack of biosynthesis could be due to transcriptional and/or translational mechanisms. On the other hand, large amounts of linamarin and lotaustralin were found in *H. sara *and *H. charithonia *which, although belonging to the same group as *H. sapho*, are not *Astrophea *specialists but *Decaloba* specialists*. *Thus, specialization for sequestration of cyclopentenyl CNglcs and feeding on woody *Astrophea* species seem to be related and could have happened in the recent radiation of the *sara‐sapho* group.

**Figure 6:**
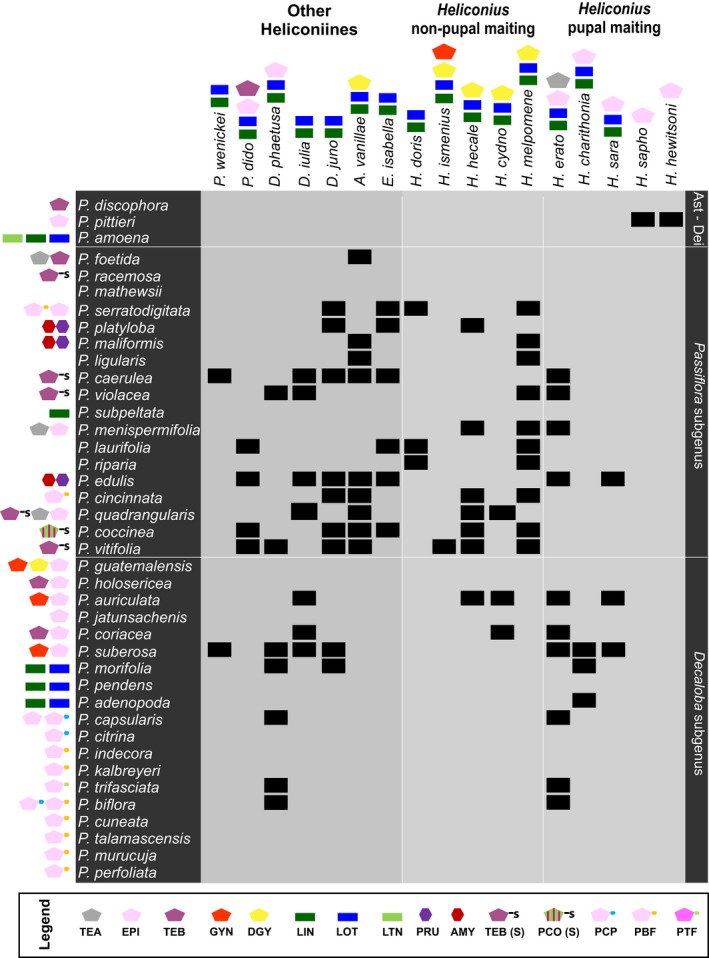
Hit‐map overlaying the host‐plant utilization by different heliconiines species described by Benson et al. (1975) and the chemical composition of the *Passiflora* species revised in this study

The fact that cyclopentenyl CNglcs were not found in *H. atthis*, *H. doris*, *E. isabella*, *D. phatethusa*, *D. juno,* and *P. wernickei* does not necessarily mean that these species lack the ability to sequester these compounds from *Passiflora*. They could simply have been feeding on *Passiflora* species devoid of cyclopentenyl CNglcs or on tissues where these compounds are not abundant to allow sequestration. Indeed, two of these species that lacked cyclopentenyl CNglcs, *H. doris *and *H. atthis,* are reported to use as larval host *P. laurifolia*, *P. riparia*, and *P. subpeltata *which do not produce these compounds (Benson et al., 1975) (Figure 6, Table 1). Since the butterflies used in this study were collected in the field or raised from pupae provided by commercial butterfly farms, their larval host plant is unfortunately unknown.

All *Heliconius *species which are *Decaloba* specialists sequestered epivolkenin. Actually, epivolkenin was found to be very common in the *Decaloba *subgenus, present in all the species analyzed, a part of the sections *Bryonioides* and *Decaloba*. Interestingly, there was no epivolkenin in the nonpupal mating *Heliconius *species, which generally contained dihydrogynocardin. Dihydrogynocardin was not common in *Passiflora* in our analyses, only present in *P. guatemalensis* which is not reported as a host of heliconiines. However, since the *Passiflora *genus has over 500 species and only 40 have had their chemical profile analyzed here, these butterflies could have fed on a species that produces dihydrogynocardin which was not present in our study. Another explanation is that nonpupal mating *Heliconius *convert epivolkenin into dihydrogynocardin, through a reduction and a hydroxylation reactions. Indeed, it has been shown that after host shifting, *H. melpomene* larvae change the expression of several P450s (Yu et al., 2016), and one of them could be involved in catalyzing this conversion. Further analyses are necessary to confirm the origin of dihydrogynocardin in these butterflies.

*Heliconius *butterflies are thought to be more toxic than basal heliconiines because they are generally more distasteful to avian predators (Chai, 1990). Cardoso and Gilbert (2013) observed that freshly emerged butterflies of *H. charithonia *have a higher cyanide emission after tissue disruption than *A. vanillae *and *D. iulia*. In our analyses, *Heliconius* species tended to have higher CNglc concentrations than other heliconiines; however, the differences were not statistically significant. Also, sequestration of cyclopentenyl CNglcs seems to be more common in *Heliconius* than in other genera. Spencer (1988) suggested that the aglycones resulting from the degradation of cyclopentenyl CNglcs were more toxic than the aglycones derived from aliphatic CNglcs, and this could contribute to the higher unpalatability of *Heliconius*.

Resource partitioning could be the reason why sequestration of CNglcs is less common in the basal heliconiines. Most of them feed on mature leaves of *Passiflora* plants (Benson et al., 1975), which as many cyanogenic plants—for example, *Sorghum bicolor* (Busk & Lindberg Møller, 2002) and *Lotus japonicus* (Forslund et al. 2004)—probably have lower concentrations of defense compounds and are nutritionally poorer than the new leaves and meristems preferred by *Heliconius* (Gilbert, 1991). Avoidance of competition with *Heliconius* larvae seems to drive larvae of *D. iulia* to leaves of lower quality (Millan, Borges, Rodrigues, & Moreira, 2013).

### 4.4 Structural diversification of CNglcs in *Passiflora*—a tool to evade heliconiine herbivory?

From the ~60 structures of CNglcs reported in plants, 27 are found in Passifloraceae and many of them are exclusive of the *Passiflora *genus. This led to the questions: What forces drove this structural diversification? Could it be associated with their coevolution with heliconiines? All CNglcs (α‐hydroxynitrile glucosides) are able to release HCN upon degradation—if cyanogenesis is the sole bioactivity of these compounds, then what is the purpose of biosynthesizing CNglcs with many different structures?

Most plants used as larval hosts by heliconiines belong to the *Decaloba* and *Passiflora* subgenera, and curiously, 80% of the CNglc structures reported in the *Passiflora* genus are in species of these two subgenera. The addition of a sulfate moiety to cyclopentenyl CNglcs and the biosynthesis of aromatic CNglcs are examples of structural diversifications of CNglc in the *Passiflora* subgenus. Additionally, some species of the *Passiflora* subgenus have become acyanogenic, presumably as a way to avoid sequestering specialists. Schappert and Shore (1999) found that many populations of *Turnera ulmifolia* are acyanogenic, most likely because the presence of CNglcs does not deter oviposition and herbivory by *E. hegesia*, its principal herbivore. Indeed, CNglc biosynthesis has high energetic costs to *T. ulmifolia*, impacting flower production and consequently reproduction, which in the absence of herbivores, will result in selection against cyanogenesis (Schappert & Shore, 2000). This could also be the case for other species in the *Passiflora* subgenus.

The diversification of CNglc structures in the *Decaloba* subgenus followed a different path than in the *Passiflora* subgenus. Aliphatic and simple cyclopentenyl CNglcs occur in the basal sections of the subgenus, whereas in the most advanced, the cyclopentenyl CNglcs are bisglycosylated with unusual sugars. Aliphatic CNglcs are present mainly in the *Bryonioides* section, which are also called the hooked trichome group and are only used as host by few heliconiine species.

Aliphatic and simple cyclopentenyl CNglcs were found in the basal subgenus *Astrophea* and *Deidamioides, *which seems to be the ancestral cyanogenic traits of the *Passiflora *genus. Even though *Astrophea* is the most basal subgenus of *Passiflora*, molecular clock analyses suggest that it was the last subgenus of *Passiflora* to diversify (Muschner, Zamberlan, Bonatto, & Freitas, 2012). Remarkably, specialization for feeding on *Astrophea* plants also happened in the advanced radiation of *Heliconius*, in the *sara‐sapho* group (Kozak et al., 2015). Perhaps, the modified CNglc composition of the *Decaloba* and *Passiflora* subgenus could have forced these *Heliconius *to change preferences to *Astrophea *species that produce simple cyclopentenyl CNglcs. It is not yet known if the modified cyclopentenyl CNglcs (sulfated and bisglycosylated) and aromatic CNglcs can be sequestered by heliconiinae larvae. Indeed, none of these compounds were found in adults of the heliconiine species analyzed.

We constructed a hit‐map with the larval host preferences of the heliconiines butterflies used in this study (Benson et al., 1975), overlaying it with the CNglc composition of the *Passiflora* plants (Figure 6) to identify if heliconiines have preference or avoidance for specific cyanogenic glucoside structures. Most *Decaloba* plants that have only bisglycosylated cyclopentenyl CNglcs seem to have escaped heliconiine herbivory, although *P. biflora* and *P. trisfasciata* which have these compounds exclusively are common hosts for many of these butterflies. *Passiflora* species with aromatic and sulfated cyclopentenyl CNglcs are also popular hosts for nonpupal mating *Heliconius* and basal heliconiines.

Despite that heliconiines are adapted to host plants with high amounts of CNglcs, modification of the structure of their aglycones and addition of unusual sugars might not deter the feeding of these butterflies. However, it could obstruct the sequestration of these compounds and thus might discourage some heliconiines to evolve preferences for the plants with modified compounds. Unfortunately, there is no obvious overlap between the host preferences of heliconiines and the CNglc profiles of *Passiflora* species. Nevertheless, there is a pattern in the distribution of modified CNglcs within the genus *Passiflora* which could have evolved in the plants to evade herbivory.

In conclusion, we demonstrated that the sequestration of cyclopentenyl CNglcs probably arose in a common ancestor of the Heliconiinae subfamily, whereas the biosynthesis of aliphatic CNglcs seems to have appeared before the divergence between butterflies and moths. In heliconiines, the profile and amount of CNglcs is related to their feeding strategy on *Passiflora *plants. In the future, it will be important to establish which CNglcs can be sequestered by heliconiine larvae in order to understand the role of cyanogenesis in the arms race between these butterflies and their *Passiflora* hosts. In addition, since many heliconiines vary their host preference according to their locality, and plants vary their metabolite profile in different environments, population studies regarding the chemical ecology of heliconiines and their *Passiflora* hosts might reveal different patterns which we could not observe in this study.

## AUTHOR CONTRIBUTIONS

The research project that resulted in this manuscript was conceptualized by EC, MZ, MC, RF, and SB. EC conducted the sample collection assisted by MC and the chemical analyses under supervision of MZ. JZ developed the phylogenetic and statistical analyses. All authors contributed to the discussion of results, hypothesis development, and writing process of this manuscript.

## Data Availability

The raw chemical data are archived at the Department of Plant and Environmental Science of Copenhagen University and is also publically available on Dryad (https://doi.org/10.5061/dryad.2r23j1q).
