# Supplementary figures and images for: Sequestration and biosynthesis of cyanogenic glucosides in passion vine butterflies and consequences for the diversification of their host plants
Source: Ecol Evol. 2019 Apr 13;9(9):5079–93. doi: 10.1002/ece3.5062 (PMC6509390; doi:10.1002/ece3.5062)

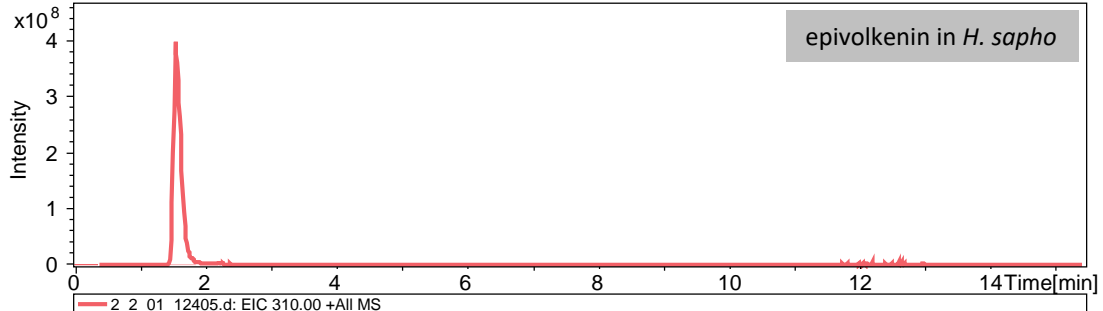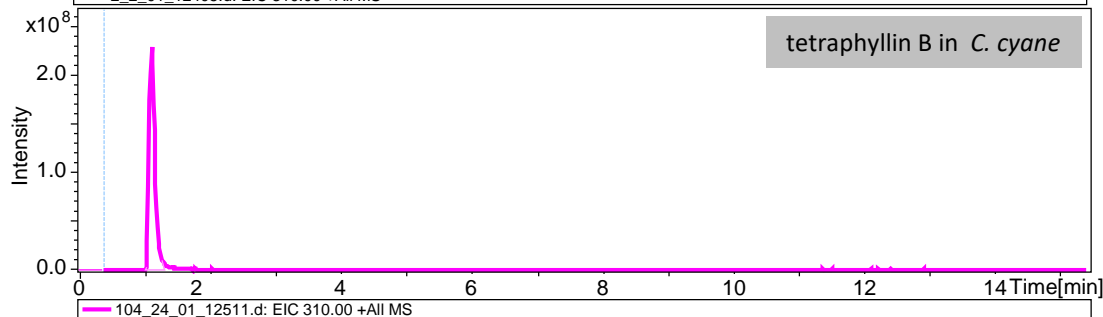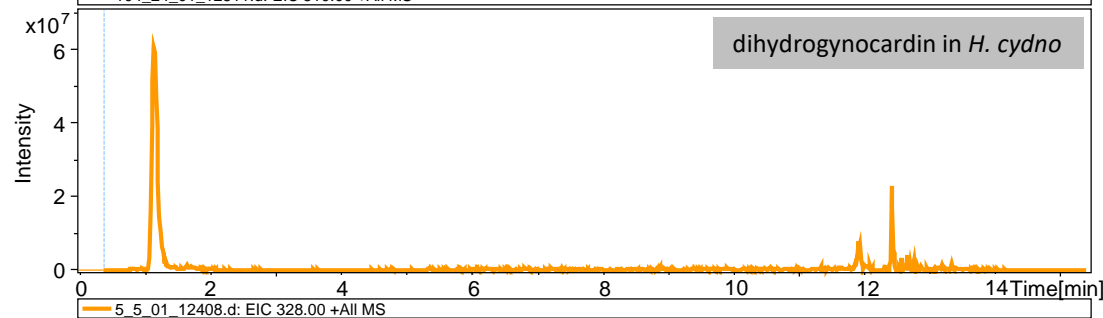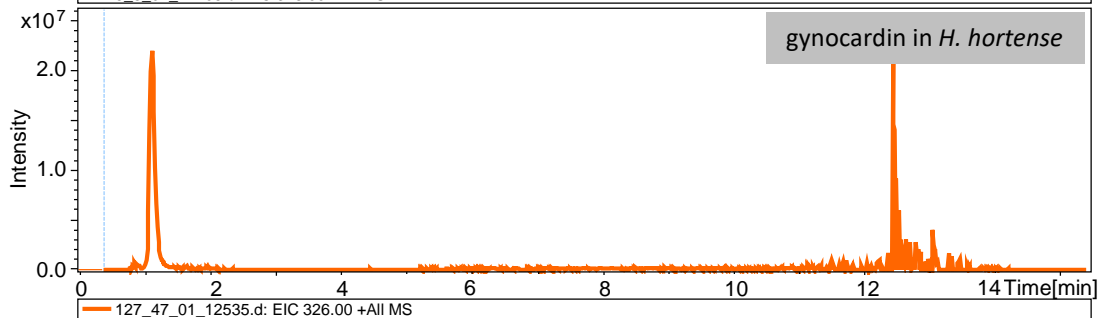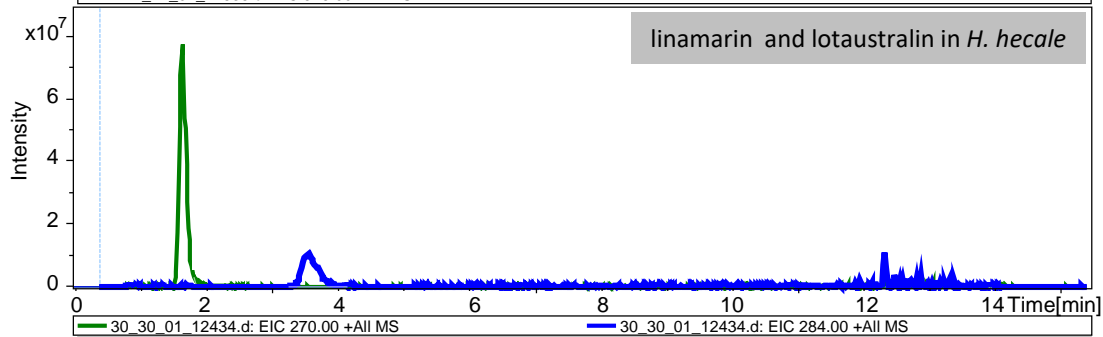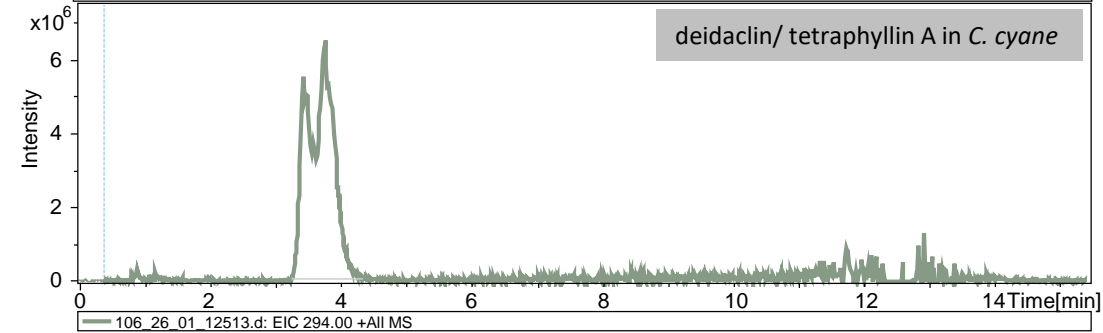

Supplement: Supplementary file 1 [file ECE3-9-5079-s001.pdf]
