# Supplementary material for: Sequestration and biosynthesis of cyanogenic glucosides in passion vine butterflies and consequences for the diversification of their host plants
Source: Ecol Evol. 2019 Apr 13;9(9):5079–93. doi: 10.1002/ece3.5062 (PMC6509390; doi:10.1002/ece3.5062)
Supplement: Supplementary file 2 [file ECE3-9-5079-s002.pdf]

Table S1. Means of total CNglc concentration in heliconiines and *Cethosia cyane*

Species	Abbrev.	*N*	CT	sd	se	ci
Cethosia cyane	Ccya	9	4.61	1.23	0.41	0.95
Dryas iulia	Diul	8	2.66	0.72	0.26	0.60
Dryadula phaethusa	Dpha	7	2.30	0.85	0.32	0.79
Dione juno	Djun	5	2.44	1.09	0.49	1.36
Agraulis vanillae	Avan	6	3.58	1.39	0.57	1.46
Eueides isabella	Eisa	3	4.17	1.18	0.68	2.93
Heliconius doris	Hdor	7	4.16	1.34	0.51	1.24
Heliconius atthis	Hatt	5	4.61	1.68	0.75	2.09
Heliconius ismenius	Hism	8	4.09	1.06	0.38	0.89
Heliconius hecale	Hhec	9	3.36	1.71	0.57	1.32
Heliconius cydno	Hcyd	10	2.67	1.03	0.33	0.74
Heliconius melpomene	Hmel	10	2.56	1.37	0.43	0.98
Heliconius erato	Hera	10	3.08	1.82	0.58	1.30
Heliconius hortenses	Hhor	4	3.41	0.84	0.42	1.33
Heliconius charithonia	Hcha	10	3.63	1.89	0.60	1.35
Heliconius sara	Hsar	10	3.09	1.18	0.37	0.84
Heliconius antiochus	Hant	4	6.17	0.78	0.39	1.25
Heliconius sapho	Hsap	9	7.45	4.78	1.59	3.68
Heliconius hewitsoni	Hhew	10	3.01	1.56	0.49	1.11

N = number of replicates. CT= means total CNglc concentrantion. Sd= standard desviation of the means

Table S2. Pairwise comparisons between total CNglc concentrations in heliconiines and *Cethosia cyane.*

	Ccya	Diul	Dpha	Djun	Avan	Eisa	Hdor	Hatt	Hism	Hhec	Hcyd	Hmel	Hera	Hhor	Hcha	Hsar	Hant	Hsap	Hhew
Ccya	-																		
Diul	0.04	-																	
Dpha	**0.01**	0.63	-																
Djun	**0.03**	0.76	0.90	-															
Avan	0.31	0.37	0.20	0.27	-														
Eisa	0.76	0.22	0.13	0.17	0.63	-													
Hdor	0.64	0.13	0.05	0.09	0.57	0.97	-												
Hatt	0.96	0.08	**0.04**	0.07	0.40	0.80	0.71	-											
Hism	0.62	0.13	0.05	0.09	0.58	0.95	0.97	0.69	-										
Hhec	0.12	0.56	0.30	0.40	0.69	0.40	0.30	0.20	0.30	-									
Hcyd	**0.02**	0.96	0.66	0.78	0.31	0.19	0.10	0.06	0.09	0.50	-								
Hmel	**0.01**	0.78	0.83	0.94	0.23	0.14	0.06	**0.04**	0.06	0.36	0.81	-							
Hera	0.06	0.75	0.42	0.54	0.51	0.29	0.18	0.12	0.19	0.77	0.68	0.52	-						
Hhor	0.32	0.46	0.28	0.35	0.93	0.60	0.55	0.39	0.57	0.79	0.42	0.32	0.61	-					
Hcha	0.21	0.34	0.16	0.24	0.95	0.56	0.48	0.31	0.49	0.70	0.27	0.18	0.49	0.97	-				
Hsar	0.09	0.64	0.34	0.46	0.60	0.35	0.24	0.15	0.23	0.89	0.57	0.43	0.86	0.70	0.60	-			
Hant	0.24	**0.01**	**0.00**	**0.01**	0.06	0.23	0.13	0.27	0.12	**0.02**	**0.01**	**0.00**	**0.01**	0.08	**0.04**	0.01	-		
Hsap	**0.04**	**0.00**	**0.00**	**0.00**	**0.01**	0.08	**0.02**	0.07	**0.01**	**0.00**	**0.00**	**0.00**	**0.00**	**0.01**	**0.00**	**0.00**	0.62	-	
Hhew	**0.03**	0.90	0.54	0.66	0.41	0.24	0.13	0.09	0.13	0.63	0.85	0.66	0.84	0.51	0.37	0.71	**0.01**	**0.00**	-

Differences statistically significant are written in bold.

Table S3. Pairwise comparisons between concentration of biosynthesized aliphatic CNglcs and sequestered cyclopentanyl CNglcs in heliconiines and *Cethosia cyane.*

	Ccya	Diul	Dpha	Djun	Avan	Eisa	Hdor	Hatt	Hism	Hhec	Hcyd	Hmel	Hera	Hhor	Hcha	Hsar	Hant	Hsap	Hhew
Ccya	-	**0.00**	**0.00**	**0.00**	**0.00**	**0.00**	**0.00**	**0.00**	**0.00**	**0.00**	**0.00**	**0.00**	**0.00**	**0.00**	**0.00**	**0.00**	**0.00**	**0.00**	**0.00**
Diul	**0.00**	-	0.98	0.98	**0.02**	0.99	0.98	0.98	0.19	0.13	**0.02**	**0.04**	0.06	0.38	0.99	**0.03**	**0.00**	**0.00**	**0.00**
Dpha	**0.00**	0.70	-	1.00	**0.02**	1.00	1.00	1.00	0.22	0.16	**0.02**	0.05	**0.04**	0.36	1.00	**0.01**	**0.00**	**0.00**	**0.00**
Djun	**0.00**	0.96	0.75	-	**0.04**	1.00	1.00	1.00	0.28	0.21	**0.04**	0.09	0.06	0.41	1.00	**0.03**	**0.00**	**0.00**	**0.00**
Avan	**0.01**	0.55	0.77	0.70	-	0.08	**0.02**	**0.04**	0.22	0.53	0.98	0.83	**0.00**	**0.02**	**0.01**	**0.00**	**0.00**	**0.00**	**0.00**
Eisa	**0.00**	0.40	0.27	0.38	0.18	-	1.00	1.00	0.42	0.35	0.10	0.18	0.13	0.53	1.00	0.07	**0.00**	**0.00**	**0.01**
Hdor	**0.00**	0.20	0.09	0.22	0.06	0.76	-	1.00	0.21	0.16	**0.02**	0.05	**0.03**	0.36	1.00	**0.01**	**0.00**	**0.00**	**0.00**
Hatt	**0.00**	0.18	0.15	0.18	0.08	0.62	0.23	-	0.28	0.22	**0.04**	0.09	0.06	0.41	1.00	**0.03**	**0.00**	**0.00**	**0.00**
Hism	**0.00**	0.30	0.44	0.31	0.23	0.32	0.07	0.38	-	0.59	0.25	0.40	**0.01**	0.36	0.16	**0.00**	**0.00**	**0.00**	**0.00**
Hhec	**0.00**	0.27	0.62	0.36	0.62	0.10	**0.02**	0.05	0.26	-	0.71	0.94	**0.00**	0.09	0.10	**0.00**	**0.00**	**0.00**	**0.00**
Hcyd	**0.01**	0.14	0.34	0.24	0.55	**0.05**	**0.00**	**0.01**	0.07	0.54	-	0.96	**0.00**	**0.02**	**0.01**	**0.00**	**0.00**	**0.00**	**0.00**
Hmel	**0.01**	0.09	0.27	0.17	0.38	**0.03**	**0.00**	**0.01**	0.08	0.57	0.78	-	**0.00**	0.05	**0.03**	**0.00**	**0.00**	**0.00**	**0.00**
Hera	**0.01**	0.09	0.26	0.17	0.38	**0.03**	**0.00**	**0.01**	0.07	0.54	0.79	1.00	-	0.07	0.02	0.95	**0.01**	**0.00**	0.23
Hhor	**0.00**	0.57	0.75	0.55	0.47	0.38	0.15	0.37	0.89	0.53	0.24	0.24	0.23	-	0.33	**0.04**	**0.00**	**0.00**	**0.01**
Hcha	**0.00**	0.10	0.20	0.13	0.10	0.17	**0.02**	0.26	0.66	0.16	**0.03**	**0.04**	**0.04**	0.57	-	**0.01**	**0.00**	**0.00**	**0.00**
Hsar	**0.01**	0.23	0.44	0.37	0.77	0.07	**0.01**	**0.02**	0.09	0.55	0.84	0.53	0.53	0.29	**0.03**	-	**0.03**	**0.00**	0.48
Hant	0.48	**0.00**	**0.00**	**0.00**	**0.00**	**0.00**	**0.00**	**0.00**	**0.00**	**0.00**	**0.01**	**0.00**	**0.01**	**0.00**	**0.00**	**0.01**	-	0.98	0.21
Hsap	0.34	**0.00**	**0.00**	**0.00**	**0.00**	**0.00**	**0.00**	**0.00**	**0.00**	**0.00**	**0.00**	**0.00**	**0.00**	**0.00**	**0.00**	**0.00**	1.00	-	**0.03**
Hhew	0.33	**0.00**	**0.00**	**0.00**	**0.00**	**0.00**	**0.00**	**0.00**	**0.00**	**0.00**	**0.00**	**0.00**	**0.00**	**0.00**	**0.00**	**0.00**	1.00	1.00	-

Differences statistically significant are written in bold.

Table S4. PGLS analysis of the correlation between CNglcs composition and larval-diet specialization in heliconiines.

	d.f	MS	R^2	F	Z	P-value
CNglc						
diet	4	0.46	0.61	5.18	3.07	<0.001
residual	13	0.08	0.38			

Table S5. PGLS analysis of the correlation between biosynthesis of aliphatic CNglcs and sequestration of cyclopentenyl CNglcs in heliconiines.

	d.f	MS	R^2	F	Z	P-value
Sequestration						
Biosynthesis	1	1.18	0.73	43.33	2.22	<0.001
residual	16	0.03	0.27			
